# Bilateral Trichotillomania of Eyelashes Triggered by Anxiety due to Nocturnal Enuresis: A Case Report

**DOI:** 10.1155/2019/4650217

**Published:** 2019-06-20

**Authors:** Shoko Ubukata, Tatsuya Mimura, Emiko Watanabe, Koichi Matsumoto, Makoto Kawashima, Kazuma Kitsu, Mai Nishio, Atsushi Mizota

**Affiliations:** Department of Ophthalmology, Teikyo University School of Medicine, Tokyo 173-8605, Japan

## Abstract

**Purpose:**

Trichotillomania is a behavioral and mental disorder and is characterized by a recurring habit of pulling out one's hair. The differential diagnosis between trichotillomania and other hair loss conditions such as alopecia areata is difficult for ophthalmologists. We report a rare case of bilateral trichotillomania of the eyelashes that was triggered by anxiety about nocturnal enuresis.

**Case Report:**

A healthy 9-year-old Japanese boy presented with a bilateral loss of his eyelashes. His parents had believed that his loss of eyelashes was due to alopecia, an autoimmune disorder that results in hair loss, of the eyelashes. Our initial examination revealed that he had suffered from nightly nocturnal enuresis from childhood and was scheduled to go on a school trip the following month. He feared that his school mates might find out about his enuresis, and he said that the anxiety was the cause of the eyelash trichotillomania. The trichotillomania was resolved by discussion among the student, his family, teacher, and school counselor.

**Conclusion:**

To the best of our knowledge, this is the first report of eyelash trichotillomania caused by anxiety about nocturnal enuresis. Ophthalmologists should be aware that a patient without eyelashes may not be due to alopecia but some anxiety-producing events. In addition, discussion of the anxiety-producing factor among the parents, teacher, and school counselors can resolve the trichotillomania.

## 1. Introduction

Trichotillomania is a hair-pulling mental disorder and is characterized by recurrent, irresistible urges to pull hair out of the body [1, 2]. Patients are fearful to tell their family and doctor, and children with trichotillomania often do not tell their parents. Thus, trichotillomania is frequently misdiagnosed as alopecia areata or other hair loss conditions on initial presentation. The most common site of hair pulling is the scalp, and it occasionally occurs for the eyelashes [3–9]. We report an unusual case of trichotillomania involving the eyelashes caused by anxiety caused by nocturnal enuresis.

## 2. Case Report

The patient was a 9-year-old boy with a history of recurrent episodes of local hair loss which was resolved within 1 to 2 months at the age of 5-6 years. The boy had never scratched his scalp or pulled his hair in front of his parents. Parents thought that the loss of the eyelashes was alopecia, and the boy was followed without receiving any treatment. No abnormalities had been detected in his hair or eyelashes at the age of 7-8 years after entering elementary school.

At the age of 9 years, his parents saw that the eyelashes of the boy were getting fewer but did not notice that he pulled out his eyelashes. His parents were concerned about their son's loss of his eyelashes and visited our hospital.

**Figure 1:**
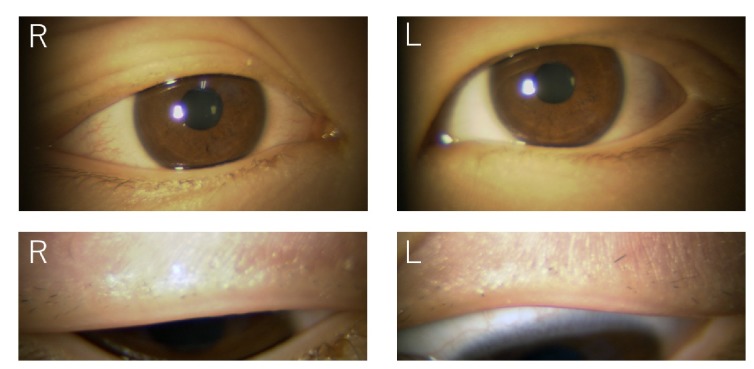
Slit-lamp photographs of an 8-year-old boy with trichotillomania of the eyelashes of both eyes. Photograph taken at initial visit.

Our examination showed that there was a complete loss of eyelashes of the upper eyelid of both eyes (Figure 1). His best-corrected visual acuity (BCVA) was 20/20 in both eyes, and his corneas were clear and did not stain with fluorescein. The anterior chamber, iris, and lens were normal. Funduscopy was within normal limits. The skin of the eyelids of both eyes were not swollen, scarred, or desquamated. The pull test of the eyelashes was negative along the edges where the eyelashes were absent and had a normal resistive response. Mycological examination of the eyelid skin was negative. These negative results of alopecia clearly confirmed the diagnosis of trichotillomania.

The mother reported that the boy had suffered from nightly nocturnal enuresis (bedwetting) all of his life, but the frequency was recently reduced to once or twice a month. However, the boy was scheduled to go on a school trip the following month, and the mother noticed that the boy had been depressed and had shown signs of avoiding school.

The boy told us that he had been concerned that his bed wetting might be revealed to his classmates during an overnight school trip. He stated that his stress was reduced by pulling out his eyelashes. We explained to the boy and his mother our diagnosis of trichotillomania and assured them that the condition could be resolved without medications. We suggested that a discussion be held among the parents, his school teacher, and school counselor about his bedwetting especially during the school trip.

One week after the school trip, the boy and mother visited our hospital and reported that no enuresis occurred during the school trip. The boy was followed without any special treatment. The mother reported that the child had not pulled out his eyelashes or hair after the consultation. With the parents' cooperation, the boy also developed control over his bladder during the night and have dry nights by age 10 years.

## 3. Discussion

Eyelash trichotillomania is extremely rare and has never been reported in a Japanese individual. A summary of the case reports on eyelash trichotillomania is presented in Table 1. In our case, the patient had a temporary stress-induced trichotillomania for 1-2 months while in kindergarten. The important causal factors for trichotillomania are stressful social or familial events such as abuse, family conflicts, or death [1, 2]. However, there were no significant stressful life events in this patient. Thus, this patient is a rare case of recurrent trichotillomania over a three-year period whose latest episode was triggered by an upcoming overnight school trip. He had great anxiety that his nocturnal enuresis will be found out by his classmates.

Trichotillomania is a mental disorder that involves irresistible urges to pull out ones hair [1, 2]. Trichotillomania can develop at all ages, and the age of the first episode of trichotillomania ranges from 9 to 13 years [10]. Our patient developed trichotillomania at the age of 5 years, and this age of onset was quite young compared to cases in general. Our patient was a young boy but there appears to be a female predominance among preadolescents to young adults and 70 to 93% of the patients are girls [11–13].

Trichotillomania can be triggered by different types of neurological disorders and mental disorders such as anxiety, depression, and obsessive–compulsive disorders [14, 15]. Young children with autism spectrum disorder (ASD) may also engage in self-stimulatory trichotillomania [16]. Our patient did not have ASD or any neurological disorders. Most of the symptoms can be resolved after reducing the anxiety, and then trichotillomania generally follows a benign course.

Most young patients with trichotillomania are managed conservatively. Scolding can be counterproductive. Thus, parents and family need to simply ignore the behavior of trichotillomania without pointing out or cautioning the child's behavior. In more severe cases, patients need to be referred to a psychologists or psychiatrists for treatment which may include medications.

In our case, the nocturnal enuresis was associated with the recurrence of trichotillomania. Nocturnal enuresis itself can be frustrating for children because parents may punish their kids for wetting the bed. However, our patient had never been scolded for his nocturnal enuresis by his parents. Thus, it appeared that the patient did not feel nocturnal enuresis itself was stressful. However, the patient was extremely vulnerable to other people's awareness of his enuresis especially his classmates. The boy was worried that his classmates would become aware of his enuresis on the school trip. Therefore, the classroom teacher, school counselor, and the parents needed to care for the children's distress, such as enuresis, throughout their school life.

In conclusion, as best we know, this is the first case report of recurrent bilateral trichotillomania involving the eyelashes triggered by anxiety of nocturnal enuresis. Punishing the child for the nocturnal enuresis can cause anguish; therefore, it is important not to scold or punish the child. Fortunately, our patient had a good outcome following conservative management. However, the management of trichotillomania with emotional distress in children at school may require strong cooperation among the school teacher, patient, and parents.

## Figures and Tables

**Table 1:** Summary of case reports of eyelash trichotillomania.

No	Age/sex (Onset age)	Country	Ocular involvement	Anxiety/ Stress	Therapy	Ocular disease	Other disease	Journal	Year	Author
1	33F	Australia	Both	Y	Behavioral therapy	None	Depression	Aust N Z J Ophthalmol	1995	Smith
2	12M	United Kingdom	Both	N	Psychiatric treatment	None	None	Br J Ophthalmol	2001	Patil
3	19F	Canada	Right	Y	Treatment of hyperthyroidism	None	hyperthyroidism	Semin Plast Surg	2007	Jordan
4	6F	Spain	Both	N	antipsychotics or mood stabilizers	None	ADHD, BD	J Can Acad Child Adolesc Psychiatry	2012	Olza-Fernández
5	55F (12)	United State of America	Both	N	Bimatoprost.	None	None	Optom Vis Sci.	2013	Peabody
6	12F	Morocco	Left	N	Cognitive - behavioral therapy	None	None	Pan African Medical Journal	2017	Touzani
7	9M (5)	Japan	Both	Y	None	None	None		This Case	Ubukata, Mimura

Age, years; M, male; F, female; Y, yes; N, no; attention-deficit/hyperactivity disorder, ADHD; pediatric bipolar disorder, BD.

## References

[1] Franklin M. E., Zagrabbe K., Benavides K. L. (2014). Trichotillomania and its treatment: a review and recommendations. *Expert Review of Neurotherapeutics*.

[2] Huynh M., Gavino A. C., Magid M. (2013). Trichotillomania. *Seminars in Cutaneous Medicine and Surgery*.

[3] Smith J. R. (1995). Trichotillomania: ophthalmic presentation. *Australian & New Zealand Journal of Ophthalmology*.

[4] Patil B. B., Dowd T. C. (2001). Trichotillomania. *British Journal of Ophthalmology*.

[5] Jordan D. R. (2007). Eyelash loss. *Seminars in Plastic Surgery*.

[6] Olza-Fernández I., Palanca-Maresca I., Jiménez-Fernández S., Cazorla-Calleja M. R. (2012). Trichotillomania, bipolar disorder and white matter hyperintensities in a six-year old girl. *Journal of the Canadian Academy of Child and Adolescent Psychiatry*.

[7] Peabody T., Reitz S., Smith J., Teti B. (2013). Clinical management of trichotillomania with bimatoprost. *Optometry and Vision Science*.

[8] Touzani K. D., Lamari Z., Chraibi F., Abdellaoui M., Andaloussi I. B. (2017). Trichotillomania involving the eyelashes: about a case. *Pan African Medical Journal*.

[9] Sławińska M., Opalska A., Mehrholz D., Sobjanek M., Nowicki R., Barańska-Rybak W. (2017). Videodermoscopy supports the diagnosis of eyelash trichotillomania. *Journal of the European Academy of Dermatology and Venereology*.

[10] Sah D. E., Koo J., Price V. H. (2008). Trichotillomania. *Dermatologic Therapy*.

[11] Christenson G. A., Mackenzie T. B., Mitchell J. E. (1991). Characteristics of 60 adult chronic hair pullers. *The American Journal of Psychiatry*.

[12] Cohen L. J., Stein D. J., Simeon D. (1995). Clinical profile, comorbidity, and treatment history in 123 hair pullers: a survey study. *Journal of Clinical Psychiatry*.

[13] Muller S. A. (1987). Trichotillomania. *Dermatologic Clinics*.

[14] Cnhristenso G. A., Crow S. J. (1996). The characterization and treatment of trichotillomania. *The Journal of Clinical Psychiatry*.

[15] Chamberlain S. R., Menzies L., Sahakian B. J., Fineberg N. A. (2007). Lifting the veil on trichotillomania. *The American Journal of Psychiatry*.

[16] Masiran R. (2018). Autism and trichotillomania in an adolescent boy. *BMJ Case Reports*.

